# Steroid-induced glaucoma in kidney transplant recipients: a prospective cross-sectional study

**DOI:** 10.22336/rjo.2024.66

**Published:** 2024

**Authors:** Vaibhav Kumar Jain, Rachna Agarwal, Lubna Maroof, Manas Ranjan Behera, Jaya Kaushik, Sushil Ojha

**Affiliations:** 1 Department of Ophthalmology, Sanjay Gandhi Post Graduate Institute of Medical Sciences, Lucknow, India; 2 Department of Nephrology, Sanjay Gandhi Post Graduate Institute of Medical Sciences, Lucknow, India; 3 Department of Ophthalmology, Command Hospital, Lucknow, India; 4 Department of Ophthalmology, Doon Medical College, Dehradun, India

**Keywords:** steroid induced glaucoma, ocular hypertension, post renal transplant, kidney transplant, cataract, ocular abnormality, glaucoma, CKD = chronic kidney disease, ESRD = end stage renal disease, OHT = ocular hypertension, IOP = intraocular pressure

## Abstract

**Objective/Aim:**

To determine the incidence of steroid induced glaucoma and treatment characteristics in kidney transplant patients in a tertiary level multispecialty institution.

**Methods:**

In this prospective cross-sectional study, the patients who underwent kidney transplant were enrolled and underwent comprehensive ophthalmological evaluation including intraocular pressure (IOP) measurement with Goldmann Applanation tonometry, visual field examination with Humphrey Field Analyzer, and gonioscopy. Cases with IOP > 21 mm Hg, visual field defect, and optic disc cupping > 0.7 or asymmetry of 0.2 or more were labeled as glaucoma, whereas IOP > 21 mm Hg with a normal visual field was designated as ocular hypertension (OHT).

**Results:**

The mean age of patients was 39 ± 9 (range: 25-60) years. Out of 72 patients with kidney transplants, 7 (9.72%) patients were diagnosed with steroid-induced glaucoma, and 9 (12.5%) patients had ocular hypertension (OHT). Four (5.55%) patients underwent trabeculectomy to control IOP whereas 3 (4.16%) patients were controlled on anti-glaucoma medications. Best-corrected visual acuity < 6/9 was noted in 23 (31.94%) patients in at least one eye. The average follow-up was 30 months with interquartile range of 18-84 months. There was a significant association of cumulative dosage of steroid and development of cataract and OHT and glaucoma (P < 0.01).

**Conclusion:**

Kidney transplant patients must be screened for glaucoma and other ocular abnormality and should be on routine ophthalmological follow-up due to the possibility of steroid induced glaucoma.

## Introduction

Globally, chronic kidney disease (CKD) poses a significant burden of morbidity and mortality. The estimated prevalence of CKD is 800 per million population, with end-stage renal disease (ESRD) at 150-200 per million population [1]. Kidney transplantation is considered the optimal therapeutic option for end-stage renal disease patients, necessitating prolonged immunosuppression for successful transplantation. These immunosuppressants mainly involve systemic steroids, cyclosporin, tacrolimus, mycophenolate mofetil, and azathioprine. Patients commonly receive combinations of these agents, which can lead to adverse reactions, including ocular manifestations.

There have been reports of posterior subcapsular cataract, steroid-induced glaucoma, retinal vein occlusion, hypertensive angiopathy, diabetic macular edema, cytomegalovirus retinitis, herpes simplex virus keratitis, herpes zoster virus keratitis, pigmentary abnormality, cystoid macular edema in patients with kidney transplant in earlier studies [2-4]. Some of the earlier studies reported a very high incidence of ocular complications (>75%) [5-7]. The ocular complications in kidney transplant patients pose a significant ocular morbidity. Prolonged immunosuppression in the form of systemic steroids is one of the factors responsible for some ophthalmic complications like cataracts and glaucoma. Steroid-induced glaucoma remains a prevalent ocular condition among kidney transplant recipients.

Nonetheless, few studies have investigated the occurrence and pattern of steroid-induced glaucoma in kidney transplant recipients. This study aims to determine the incidence of steroid-induced glaucoma, as well as its treatment characteristics, in kidney transplant recipients within a tertiary care multispecialty institute in the central part of India.

## Methods

This prospective cross-sectional study enrolled consecutive patients undergoing routine follow-up in the Nephrology Department for 1 year. The study adhered to the Declaration of Helinski, with ethical clearance from the Institutional Ethics Committee (2020-335-IMP-EXP-34).

Patients followed the institute’s protocol for immunosuppressant regimens, consisting of induction and maintenance phases. The induction regimen consisted of an injection of Methylprednisolone (1 gm single dose in Operation Theater before arterial anastomosis) and either Basiliximab (20 mg on day 0 and day 4) or anti-thyroglobulin (1.5 mg/kg/day for three doses on day-0, 3 and 5). The maintenance regimen consisted of a triple immunosuppressant of Steroids, CNI (Tacrolimus), and antimetabolites (Mycophenolate), which started 2 days before the transplant and continued for a lifetime. Steroids were started at a dose of 20 mg/day. After the 2nd month, the steroid was tapered gradually by 2.5 mg/week to achieve 10 mg/day by the end of the 3rd month. Tacrolimus was started at a dose of 0.1 mg/kg/day in two divided doses and was tittered to achieve a whole blood trough level of 10-12, 8-10, 6-8, and 4-6 ng/ml in the 1st, 2-3rd, 4-6th and after 6th months of transplant, respectively. Mycophenolate Mofetil/Sodium was initially started at doses 1 gm/720 mg twice daily, respectively, and doses were tittered to maintain a total leucocyte count of more than 4000 cells per cubic millimeter of blood.

All patients then underwent comprehensive ophthalmic evaluation involving visual acuity (Snellen Chart), anterior segment examination with slit lamp biomicroscopy, and fundus evaluation with +90 D stereoscopic examination using a slit lamp. Intraocular pressure (IOP) was measured with a Goldmann Applanation tonometer. Gonioscopy and Visual field (Humphrey Field Analyzer) examination with central 30-2 were also performed. Steroid-induced glaucoma was labeled in cases with IOP > 21 mm Hg, visual field defect, optic disc cupping > 0.7, asymmetry of 0.2 or more, and open-angle on gonioscopy. In contrast, patients with IOP > 21 mm Hg with normal visual field and open-angle on gonioscopy were designated as ocular hypertension (OHT). Patients with pre-existing glaucomatous optic neuropathy, cataract, and occludable angles were excluded. Other associated ocular disorders were also noted in these patients. A total number of days with steroids was assessed for any relation to the development of cataracts and glaucoma. A cumulative dose of steroids was calculated at the time of examination. Also, the number of days for which patients were on high dosages of steroids (> 100 mg per day) for rejection was calculated. Both the cumulative dose and number of days of high steroids were assessed for any relation to the development of cataracts and glaucoma.

Data was spread in an Excel sheet, and the number of patients with ocular abnormalities in at least one eye was counted. SPSS software was used for statistical analysis. Using the Chi-square test and unpaired “t” test, a relationship was studied between the dosage, duration, and number of days with high-dose steroids for cataracts, ocular hypertension, and glaucoma.

## Results

The study enrolled 72 kidney transplant patients. The mean age of patients was 39 ± 9 (range: 25-60) years. **Table 1** describes the various demographic characteristics of study patients. **Table 2** shows the etiological diagnosis of patients with chronic kidney disease or end-stage renal disease.

**Table 1:** Demographic characteristics of study patients

Characteristics	Values
Total no. of patients enrolled	72
Mean age (± SD) years	39 (± 9) (Range: 25-60 years)
Sex (Male/Female)	58/14
Follow-up period of kidney transplantation median (months)	30 months (Range: 18-84 months)

**Table 2:** Various etiology of chronic kidney disease/end-stage renal disease

Etiology	Number of patients
Diabetic nephropathy	31
Chronic glomerulonephritis	16
Tubulointerstitial nephritis	7
Idiopathic	18

Steroid-induced glaucoma was diagnosed in 7 patients (9.72%), while ocular hypertension was observed in 9 patients (12.5%), contributing to 16 patients (22.22%) with steroid-related-glaucoma disorders. The cataract incidence was 52.77% (38 patients), with 8 (11.11%) patients undergoing surgery with intraocular lens implantation. Other ocular findings are described in **Table 3**. The clinical characteristics of patients with ocular hypertension and steroid-induced glaucoma are described in **Table 4**. Patients with steroid-induced glaucoma had more advanced cupping (0.7 ± 019) and greater IOP (30.2 ± 6.38 mm Hg) than patients with ocular hypertension [cup: disc ratio 0.4 (± 0.07); IOP 22.2 (± 0.80) mm Hg]. In steroid-related glaucoma disorders, three patients (4.16%) required anti-glaucoma medications to control IOP, whereas four patients (5.55%) required trabeculectomy for further control of IOP. Diminished best-corrected visual acuity (< 6/9) was observed in 23 patients (31.94%) in at least one eye.

**Table 3:** Various ocular disorders in patients with kidney transplants

Diagnosis	Number of patients (%)
Steroid-induced glaucoma	7 (9.72%)
Ocular hypertension	9 (12.5%)
Cataract	38 (52.77%)
Hypertensive retinopathy	10 (13.88%)
Diabetic retinopathy	4 (5.55%)
Branch retinal vein occlusion	1 (1.38%)

**Table 4:** Characteristics of patients with steroid-induced glaucoma and ocular hypertension

Characteristics	Ocular hypertension	Steroid-induced glaucoma
Mean age (± SD) years	44 (11.49)	39 (2.28)
Sex ratio (male/female)	6/3	5/2
Follow-up of kidney transplant median (months) range	19 (3-36)	18 (6-72)
Mean (± SD) IOP mm Hg	22.2 (0.80)	30.2 (6.38)
Mean (± SD) cup: disc ratio	0.4 (0.07)	0.7 (0.19)
Number of patients with anti-glaucoma medications	2	1
Number of patients with trabeculectomy	-	4

IOP = intraocular pressure

Dose-dependent relation was assessed for patients with or without cataracts and steroid-induced OHT/Glaucoma (**Table 4**). There was no statistically significant difference between the patients with or without cataracts for the total number of days they were on steroids. Similarly, the total number of days of steroid medication was not found to be significantly different between the patients who had glaucoma and those who did not have glaucoma (P > 0.05). However, the patients with cataract and steroid-induced OHT and glaucoma were found to have received a significantly higher amount of cumulative dosage of steroids (P < 0.01) (**Table 5**). The number of days with a high dosage of steroids (> 100 mg per day) was also assessed to find out any further relation between the development of cataracts and glaucoma and high steroid dosage (**Table 5**). For a highly significant number of days, patients who received high dosages of steroids developed cataracts and glaucoma.

**Table 5:** Relation between steroid dose and steroid-induced cataract/glaucoma

	Glaucoma and ocular hypertension (Yes)	Glaucoma and ocular hypertension (No)	P Value
Total number of days (days)	1020	972	0.981*
Total dosage (g)	31.45	23.78	< 0.01**
High dosage received for days (> 100 mg/day)	25	15	< 0.01*
	Cataract (Yes)	Cataract (No)	
Total number of days	1120	1012	0.872*
Total dosage (g)	34.32	25.76	< 0.01**
High dosage received for days (> 100 mg per day)	20	9	< 0.01*

* Chi Square test; **Unpaired t test; P < 0.05 significant

## Discussion

Steroids are widely used for treating ocular and systemic conditions, often causing side effects with prolonged usage. They are one of the most commonly prescribed drugs for various chronic conditions. Long-term administration of drugs for some chronic conditions like autoimmune diseases and post-transplant recipients might lead to the possibility of multiple side effects. Therefore, the patients must be monitored closely to rule out any possible complication related to the prolonged use of steroids. Steroid-related ocular complications mainly arise from topical steroids and periocular steroids. However, they are also found in patients with protracted usage of systemic steroids [8].

Steroid-induced glaucoma is a form of open-angle glaucoma. Various possible mechanisms of steroid-induced glaucoma in such patients include stabilization of the lysosomal membrane and accumulation of polymerized glycosaminoglycans in the trabecular meshwork, which causes biologic edema with consequent outflow obstruction [9]. Other mechanisms include increased extracellular matrix protein expression and glucocorticoid receptor beta expression via FK506 binding protein 51 [10].

The incidence of ocular complications varied in different series. We report ocular complications in 38 (52.77%) patients of kidney transplant recipients. A case series from India has reported the incidence of ocular abnormality in 52.5% of patients [11]. Other series have reported a higher incidence of ocular complications as 80.4% [7], 77.8% [5], 75.5% [6], and 74% [12]. In our series, cataract was the most common ophthalmic complication encountered in 52.77% of patients. The incidence of cataracts varied from 23% to 62.5% in earlier patients receiving renal transplants [2-7,11-15].

Ocular hypertension was the next most common steroid-related ophthalmic complication encountered in 12.5% of our patients. Similarly, ocular hypertension has been reported in 12.5% of patients in a study by Shimmyo et al. [5]. Six (9.67%) patients out of 62 developed ocular hypertension in a series by Adhikary et al. [13]. The high incidence of ocular hypertension has been reported as 47%, with one eye developing glaucomatous changes in a series of 15 patients of kidney transplant recipients [15]. A small number of patients in this series might be one factor for reporting a high incidence of ocular hypertension [12]. A long-term corticosteroid was the factor responsible for the IOP being raised. In another series, only one patient developed glaucomatous cupping and increased IOP responded well to the topical anti-glaucoma medications [14]. Another series reported only two cases of open-angle glaucoma in 104 kidney transplant patients, indicating a minimal risk of steroid-induced glaucoma [16]. A recently published 10-year analysis of eyesight in 50 eyes of 25 patients after kidney transplantation revealed cataract (48%), hypertensive retinopathy (28%), diabetic macular edema (16%), and glaucoma (16%) as an ophthalmological complication [4]. We had 7 (9.72%) patients with steroid-induced glaucomatous cupping and visual field defect, among whom four patients had trabeculectomy, and three patients required antiglaucoma medications. We observed hypertensive retinopathy in 13.88%, diabetic retinopathy in 5.55%, and one case of branch retinal vein occlusion.

A relation between the development of cataract/glaucoma and duration of steroid therapy, cumulative dosage, and number of days of high dosage of steroid (> 100 mg per day) was assessed in our study. Patients who developed cataract/glaucoma were more likely to receive higher cumulative dosages of steroids and more days of high dosages of steroids (> 100 mg per day). Similar associations between the development of cataracts and steroids have been found in a previous study involving 62 renal transplant patients by Pavlin et al. [14]. They also found a significant correlation between the development of cataracts and the number of rejection episodes. Porter et al. also observed a correlation between cataracts and the total steroid dosage [2].

## Conclusion

In conclusion, regular ophthalmological screening is crucial for kidney transplant recipients as early detection and management of ocular complications can help preserve or improve vision.
